# Cardiologia Nuclear na Otimização da Terapia de Ressincronização: Quo Vadis?

**DOI:** 10.36660/abc.20230136

**Published:** 2023-03-27

**Affiliations:** 1 Hospital Moinhos de Vento Medicina Nuclear Porto Alegre RS Brasil Hospital Moinhos de Vento – Medicina Nuclear, Porto Alegre, RS – Brasil

**Keywords:** Medicina Nuclear, Cardiologia, Radioisótopos, Isquemia Miocárdica, Imagem de Perfusão Miocárdica, Diagnóstico por Imagem/métodos, Insuficiência Cardíaca, Terapia por Ressincronização Cardíaca

A Cardiologia Nuclear tem papel de destaque na avaliação e definição da conduta do paciente cardiopata.^1^ É um método estabelecido na investigação e acompanhamento de pacientes com cardiopatia isquêmica (CI) suspeita ou estabelecida, através da cintilografia de perfusão miocárdica sincronizada com eletrocardiograma (GATED-SPECT) ou tomografia por emissão de pósitrons (PET), para a determinação de isquemia e viabilidade miocárdica e desempenha um papel cada vez mais importante na investigação da disfunção microvascular.

Muito além da CI, a Cardiologia Nuclear é cada vez mais útil na avaliação de doenças infiltrativas, como sarcoidose e amiloidose, e infecciosas, como endocardite infecciosa, e tem papel potencial no acompanhamento de pacientes com suspeita de cardiotoxicidade.

Em pacientes com insuficiência cardíaca (IC), além da investigação de sua etiologia, que se refere às indicações acima, é útil, embora subutilizada, na avaliação do sistema nervoso autônomo para indicar a colocação de cardioversor/desfibrilador implantável (CDI) e tem um enorme potencial para otimizar a terapia de ressincronização cardíaca (TRC), considerando que uma porcentagem significativa de pacientes que atendem aos critérios de indicação dessa terapia não responde conforme o esperado. Nesse sentido, a Cardiologia Nuclear pode ajudar a otimizar a TRC avaliando a viabilidade miocárdica no local de implantação do eletrodo, a dissincronia ventricular e a localização do último segmento a se contrair.^2^

## Viabilidade Miocárdica e Terapia de Ressincronização

Vários estudos na literatura mostram que a ausência de viabilidade miocárdica no local onde o eletrodo foi posicionado reduz a eficácia da TRC. Ypenburg et al.,^3^ avaliaram a presença de fibrose usando GATED-SPECT com Tc-99m-tetrofosmin antes da TRC. Pacientes sem fibrose no local de implantação do eletrodo apresentaram melhora na classe NYHA, qualidade de vida, teste de caminhada de 6 minutos, volumes ventriculares e fração de ejeção em 6 meses de seguimento em comparação com pacientes com fibrose no local de implantação do eletrodo para TRC. A extensão da área miocárdica viável foi relacionada à redução dos volumes ventriculares e da fração de ejeção.^3^ Bose et al.^4^ também demonstraram em um estudo retrospectivo envolvendo 160 pacientes com cardiomiopatia isquêmica que aqueles pacientes em que a colocação do eletrodo estava em um local com fibrose ou fibrose e isquemia tiveram mais frequentemente o desfecho primário (hospitalização por IC e morte) em 3 anos em comparação com aqueles pacientes com miocárdio normal. Portanto, a avaliação de fibrose e viabilidade miocárdica no local de implantação do eletrodo deve ser considerada antes da TRC.^4^

## Avaliação da dissincronia ventricular e localização do último segmento a contrair

Inicialmente obtido por ventriculografia radioisotópica, o sincronismo ventricular por análise de fase pode atualmente ser analisado por meio da cintilografia miocárdica. Um método baseado em contagem permite a extração da amplitude (que reflete o espessamento sistólico da parede) e da fase das alterações regionais da contagem do ventrículo esquerdo ao longo do ciclo cardíaco.^5^ Essa técnica permite a análise da dissincronia ventricular e a detecção do último segmento que se contraiu no ciclo cardíaco. A possibilidade de analisar o sincronismo cardíaco através do GATED-SPECT foi, sem dúvida, um avanço que possibilitou a análise desses dados na rotina de um laboratório de Cardiologia Nuclear.

Boogers et al.,^6^ avaliaram 90 pacientes com IC e indicação de TRC. Em 52 pacientes (58%), o eletrodo ventricular esquerdo (VE) foi posicionado no local da última ativação mecânica (concordante) e em 38 pacientes (42%), o eletrodo VE foi posicionado fora do local da última ativação mecânica (discordante). A resposta à TCR foi significativamente mais frequentemente documentada em pacientes com posição concordante do eletrodo VE do que em pacientes com posição discordante do eletrodo VE (79% vs. 26%, p<0,01). Após 6 meses, os pacientes com posição concordante do eletrodo VE apresentaram melhora significativa na fração de ejeção do ventrículo esquerdo, volume sistólico final VE e volume diastólico final VE.^6^ Resultados semelhantes foram obtidos por Zhang et al.,^7^ que demonstraram que a implantação do eletrodo no último segmento a se contrair, excluindo os locais com fibrose, teve benefício prognóstico.^7^ Recentemente, Peix et al.,^8^ demonstraram em um estudo multicêntrico avaliando 195 pacientes submetidos à avaliação do dissincronismo cardíaco antes da TRC que a melhora do sincronismo cardíaco, mas não o posicionamento correto do eletrodo, foi o preditor de desfechos clínicos em seguimento de 6 meses.^8^ Outro fator que deve ser considerado na análise desses estudos é a dificuldade em posicionar corretamente o eletrodo. Nascimento et al.,^9^ avaliando um pequeno grupo de pacientes, demonstraram que o posicionamento adequado do eletrodo na contração do último segmento foi possível em apenas 54% dos pacientes.^9^

Nos Arquivos Brasileiros de Cardiologia, Nascimento et al.,^10^ avaliaram a viabilidade do implante de eletrodo VE guiado por análise de fase e sua relação com o remodelamento ventricular. Em uma amostra pequena, 18 pacientes com indicação de TRC realizaram cintilografia miocárdica para orientação do implante, avaliando-se os parâmetros de excentricidade e forma ventricular. O eletrodo VE para TRC foi posicionado concordante, adjacente e discordante em 11 (61,1%), 5 (27,8%) e 2 (11,1%) pacientes, respectivamente. Excentricidade no final da sístole e da diástole demonstrou remodelamento reverso pós-TRC. Os autores concluíram que o implante de eletrodo VE para TRC guiado por cintilografia GATED SPECT é factível. A colocação do eletrodo concordante ou adjacente ao último segmento a contrair foi determinante para o remodelamento reverso.^10^

O presente estudo de Nascimento et al.,^10^ agrega informações à literatura sobre esse tema, cujo potencial é enorme para auxiliar na melhor seleção de pacientes que necessitam de terapia de ressincronização. No entanto, os dados disponíveis na literatura, embora demonstrem a viabilidade do uso do GATED SPECT para guiar a TRC com bons resultados de desfechos clínicos, apresentam uma importante limitação: o pequeno número de pacientes estudados. Estudos randomizados com um número maior de pacientes ainda são necessários para definir o papel da Cardiologia Nuclear neste cenário clínico e para onde estamos indo neste sentido.
