# Supplementary material for: Cardiologia Nuclear na Otimização da Terapia de Ressincronização: Quo Vadis?
Source: Arq Bras Cardiol. 2023 Mar 27;120(3):e20230136. [Article in Portuguese] doi: 10.36660/abc.20230136 (PMC10263451; doi:10.36660/abc.20230136)
Supplement: Translation

# Translation: Nuclear Cardiology in the Optimization of Resynchronization Therapy: Quo Vadis?

**Affiliations:** 1 Porto Alegre RS Brazil Hospital Moinhos de Vento – Medicina Nuclear, Porto Alegre, RS – Brazil

**Keywords:** Nuclear Medicine, Cardiology, Radioisotopes, Myocardial Ischemia. Myocardial Perfusion Imaging, Diagnostic, Imaging/methods, Heart Failure, Cardiac Resynchronization Therapy

Nuclear Cardiology plays a prominent role in evaluating and defining the conduct of patients with heart disease.^1^ It is a method established in the investigation and follow-up of patients with ischemic heart disease (IC) suspected or established through myocardial perfusion scintigraphy synchronized with an electrocardiogram (GATED-SPECT) or positron emission tomography (PET) in the determination of ischemia and myocardial viability and plays an increasingly important role in the investigation of microvascular dysfunction.

Far beyond IC, Nuclear Cardiology is increasingly useful in evaluating infiltrative diseases, such as sarcoidosis and amyloidosis, and infectious diseases, such as infectious endocarditis, and has a potential role in the follow-up of patients with suspected cardiotoxicity.

In patients with heart failure (HF), in addition to the investigation of its etiology, which refers to the above indications, it is useful, although underutilized in the evaluation of the autonomic nervous system to indicate the placement of implantable cardioverter/defibrillator (ICD) and has a huge potential in optimizing cardiac resynchronization therapy (CRT), considering that a significant percentage of patients who meet indication criteria for this therapy do not respond as expected. In this respect, Nuclear Cardiology can help optimize CRT by evaluating myocardial viability at the electrode implantation site, ventricular dyssynchrony, and location of the last segment to contract.^2^

## Myocardial Viability and Resynchronization Therapy

Several studies in the literature have shown that the absence of myocardial viability at the site where the electrode was positioned reduces the efficacy of CRT. Ypenburg et al.^3^ evaluated the presence of fibrosis using GATED-SPECT with Tc-99m-tetrofosmin before CRT. Patients without fibrosis at the electrode implantation site showed improvement in NYHA class, quality of life, 6-minute walk test, ventricular volumes, and ejection fraction at a 6-month follow-up compared to patients with fibrosis at the electrode implantation site for CRT RT. The extent of the viable myocardial area was related to the reduction of ventricular volumes and ejection fraction.^3^ Bose et al.^4^ also demonstrated in a retrospective study involving 160 patients with ischemic cardiomyopathy that those patients in which electrode placement was in a site with fibrosis or fibrosis and ischemia more often had the primary outcome (hospitalization for HF and death) at 3 years compared to those patients with normal myocardium. Therefore, the fibrosis and myocardial viability evaluation at the electrode implementation site should be considered before CRT.^4^

## Evaluation of ventricular dyssynchrony and location of the last segment to contract

Initially obtained through radioisotopic ventriculography, ventricular synchronism through phase analysis can currently be analyzed using myocardial scintigraphy. A count-based method allows extraction of amplitude (which reflects systolic wall thickening) and phase from the regional left ventricular (LV) count changes throughout the cardiac cycle.^5^ This technique allows the analysis of ventricular dyssynchrony and the detection of the last segment that contracted in the cardiac cycle. The possibility of analyzing cardiac synchronism through GATED-SPECT was undoubtedly an advance that made it possible to analyze this data in the routine of a Nuclear Cardiology laboratory.

Boogers et al.^6^ evaluated 90 patients with HF and indication for CRT. In 52 patients (58%), the LV lead was positioned at the site of the latest mechanical activation (concordant), and in 38 patients (42%), the LV lead was positioned outside the site of the latest mechanical activation (discordant). CRT response was significantly more often documented in patients with a concordant LV lead position than in patients with a discordant LV lead position (79% vs. 26%, p<0.01). After 6 months, patients with a concordant LV lead position showed significant improvement in LV ejection fraction, LV end systolic volume and LV end diastolic volume.^6^ Similar results were obtained by Zhang et al.,^7^ who demonstrated that the electrode implantation in the last segment to contract, excluding sites with fibrosis, had prognostic benefit.^7^ Recently, Peix et al.^8^ demonstrated in a multicenter study evaluating 195 patients submitted to cardiac dyssynchronism assessment before CRT that the improvement of cardiac synchronism, but not the correct positioning of the electrode, was the predictor of clinical outcomes in a 6-month follow-up.^8^ Another factor that should be considered in the analysis of these studies is the difficulty in correctly positioning the electrode. Nascimento et al.,^9^ evaluating a small group of patients, demonstrated that properly positioning the electrode in the last segment contracting was possible in only 54% of patients.^9^

In the *Arquivos Brasileiros de Cardiologia*, Nascimento et al.^10^ evaluated the feasibility of implantation LV lead guided by phase analysis and its relationship to ventricular remodeling. In a small sample, 18 patients with an indication for CRT performed myocardial scintigraphy for implant orientation, and eccentricity and ventricular shape parameters were evaluated. The CRT LV lead was positioned concordant, adjacent, and discordant in 11 (61.1%), 5 (27.8%), and 2 (11.1%) patients, respectively. End-systolic and end-diastolic eccentricity demonstrated reverse remodeling post-CRT. The authors concluded that LV lead implantation in CRT guided by GATED SPECT scintigraphy is feasible. The electrode placement concordant or adjacent to the last segment to contract was a determinant of reverse remodeling.^10^

The present study by Nascimento et al.^10^ aggregates information in the literature on this topic with enormous potential to help better select patients who need to undergo resynchronization therapy. However, the data in the available literature, although they demonstrate the feasibility of using GATED SPECT to guide RT with good results in clinical outcomes, have an important limitation: the small number of patients studied. Randomized studies with a larger number of patients are still needed to define the role of Nuclear Cardiology in this clinical scenario and where we are going.
